# Biosynthetic gene clusters with biotechnological applications in novel Antarctic isolates from *Actinomycetota*

**DOI:** 10.1007/s00253-024-13154-x

**Published:** 2024-05-08

**Authors:** Pablo Bruna, Kattia Núñez-Montero, María José Contreras, Karla Leal, Matías García, Michel Abanto, Leticia Barrientos

**Affiliations:** 1 Programa de Doctorado en Ciencias mención Biología Celular y Molecular Aplicada, Universidad de La Frontera, Temuco, Chile; 2 Núcleo Científico y Tecnológico en Biorecursos (BIOREN), Universidad de La Frontera, Avenida Francisco Salazar, 01145 Temuco, Chile; 3 Facultad de Ciencias de la Salud, Instituto de Ciencias Aplicadas, Universidad Autónoma de Chile, Avenida Alemania 1090, Temuco, Chile; 4 Centro de Investigación en Biotecnología, Departamento de Biología, Instituto Tecnológico de Costa Rica, Cartago, Costa Rica; 5 Facultad de Ingeniería, Instituto de Ciencias Aplicadas, Universidad Autónoma de Chile, Avenida Alemania 1090, Temuco, Chile; 6 Biocontrol Research Laboratory, Facultad de Ciencias Agropecuarias y Medioambiente, Universidad de La Frontera, Temuco, Chile

**Keywords:** Actinomycetota, Biosynthetic gene clusters, Antarctic, Secondary metabolites, *Micrococcaceae*

## Abstract

**Abstract:**

*Actinomycetota* have been widely described as valuable sources for the acquisition of secondary metabolites. Most microbial metabolites are produced via metabolic pathways encoded by biosynthetic gene clusters (BGCs). Although many secondary metabolites are not essential for the survival of bacteria, they play an important role in their adaptation and interactions within microbial communities. This is how bacteria isolated from extreme environments such as Antarctica could facilitate the discovery of new BGCs with biotechnological potential. This study aimed to isolate rare *Actinomycetota* strains from Antarctic soil and sediment samples and identify their metabolic potential based on genome mining and exploration of biosynthetic gene clusters. To this end, the strains were sequenced using Illumina and Oxford Nanopore Technologies platforms. The assemblies were annotated and subjected to phylogenetic analysis. Finally, the BGCs present in each genome were identified using the antiSMASH tool, and the biosynthetic diversity of the *Micrococcaceae* family was evaluated. Taxonomic annotation revealed that seven strains were new and two were previously reported in the NCBI database. Additionally, BGCs encoding type III polyketide synthases (T3PKS), beta-lactones, siderophores, and non-ribosomal peptide synthetases (NRPS) have been identified, among others. In addition, the sequence similarity network showed a predominant type of BGCs in the family *Micrococcaceae*, and some genera were distinctly grouped. The BGCs identified in the isolated strains could be associated with applications such as antimicrobials, anticancer agents, and plant growth promoters, among others, positioning them as excellent candidates for future biotechnological applications and innovations.

**Key points:**

*• Novel Antarctic rare Actinomycetota strains were isolated from soil and sediments*

*• Genome-based taxonomic affiliation revealed seven potentially novel species*

*• Genome mining showed metabolic potential for novel natural products*

**Supplementary Information:**

The online version contains supplementary material available at 10.1007/s00253-024-13154-x.

## Introduction

*Actinomycetota* have attracted increasing scientific interest in the vast field of microorganisms owing to their importance in the production of biologically and pharmaceutically relevant natural products (Matsumoto and Takahashi 2017; Albarano et al. 2020). Natural products derived from microorganisms have a broad spectrum of biological activities, making them promising candidates for the development of new drugs. However, the discovery of new bioactive compounds poses a great challenge to the scientific community due to the need to identify new sources of bioactive compounds with antimicrobial potential (Kumar et al. 2020; Núñez-Montero et al. 2019). Within the phylum *Actinomycetota*, *Micrococcaceae* plays a key role in the synthesis of bioactive compounds with a wide range of therapeutic properties. Exploration of these microorganisms and their natural products has opened new perspectives to address public health challenges (Borker et al. 2021; Núñez-Montero et al. 2019), such as the increasing antimicrobial resistance observed in pathogenic species.

Antarctica and its extreme environment have emerged as promising sources for the discovery of extraordinary taxa (Benaud et al. 2021) and the exploration of novel natural products (Silva et al. 2020). The extreme environmental conditions present, such as low temperatures, high radiation, and nutrient scarcity, have driven the development of unique adaptations in Antarctic microorganisms, contributing to richer biodiversity and high potential for bioactive compound discovery (Gummerlich et al. 2020). *Actinomycetota*, in particular, has been the subject of great attention in Antarctica because of their ability to produce secondary metabolites with pharmacological and biotechnological properties (Oyedoh et al. 2023) (Reis-Mansur et al. 2019). In recent years, Antarctic bacteria have been found to produce plant growth promoters, pigments, bioremediation agents, bioactive compounds, antibiofilm agents, nanoparticles, and enzymes for the food industry (Styczynski et al. 2022; Ramasamy et al. 2023).

On the other hand, advancements in DNA sequencing technology have revolutionized the study of microorganisms and their biosynthetic capabilities (Singh et al. 2021). Next-generation sequencing and related technologies have significantly accelerated and improved access to genetic information on microorganisms (Scherlach and Hertweck 2021), aiding the swift and accurate identification of biosynthetic gene clusters responsible for the production of natural products. These bioinformatics tools play a crucial role in the discovery (Tizabi et al. 2022) and characterization of new bioactive compounds (Albarano et al. 2020; Benaud et al. 2022) as well as in understanding the underlying molecular mechanisms of their biosynthesis.

While there is no certainty regarding the expression or silencing of the gene clusters identified through genomics, this initial exploration enabled us to detect their presence and structure. Mechanisms for the activation and overexpression of these genes could be proposed based on genome mining to obtain novel bioactive molecules. Therefore, genomic exploration of emerging microorganisms and their biosynthetic gene clusters lays the foundation for advanced metabolomics studies and characterization of novel biomolecules (Javed et al. 2021). In this context, the objective of our study was to isolate rare *Actinomycetota* strains from Antarctic soil and sediment samples and identify their metabolic potential based on genome mining and exploration of biosynthetic gene clusters.

## Material and methods

### Isolation and sample treatment of actinobacterial strains

In this study, soil and sediment samples were collected from Antarctica during the Chilean Antarctic Expedition conducted between 2019 and 2020. For isolation of rare *Actinomycetota* strains (less frequently isolated species), the samples were treated with high temperature, detergent solutions and plated with antibiotics supplementation to avoid the growth of most frequent species in the soil. To do this, 1 g of each sample was exposed to 100 °C for 60 min, followed by a 10^−1^ dilution in each of the following chemical treatments: 1.5% phenol, 0.05% SDS, or 1% chloramine-T, incubating for 30 min at 30 °C. A 0.85% solution was used for the control samples. Dilutions of 10^−2^ and 10^−3^ of each pre-treated sample were cultured on soil agar (100 g/L Antarctic soil, 18 g/L agar) and oatmeal agar (60 g/L oatmeal, 18 g/L agar) supplemented with nalidixic acid (25 μg/mL). The cultures were incubated for 4 weeks at 15 °C. The resulting colonies were isolated and subsequently plated until purification in ISP-2 agar medium (4 g/L yeast extract, 4 g/L glucose, 10 g/L malt extract, and 20 g/L agar). Pure cultures were preserved by freezing at −80 °C in glycerol (20% v/v) until further use.

### Culture of actinobacterial strains

In this work, nine isolated strains deposited at the Colección Chilena de Cultivos Tipo – CCCT (Universidad de La Frontera, Chile) under accession codes CCCT 24.01–CCCT 24.09 were utilized. These strains were inoculated on ISP-2 agar plates, nutrient agar (Liofilchem, Italy), and R2A agar (Merck Millipore, Germany) and incubated at 15 °C for 2 weeks until suitable microbial growth was observed.

### DNA extraction from actinobacterial strains

From the above cultures, DNA extraction was performed using DNeasy UltraClean Microbial Kit (QIAGEN, Germany) following the manufacturer’s instructions. Subsequently, DNA was quantified using a Qubit dsDNA HS Assay Kit (Invitrogen, USA), considering a minimum range of concentrations equal to or greater than 50 ng/µL. Finally, the integrity of the obtained DNA was visualized on an agarose gel in TAE 1 × 0.9% m/v buffer.

### Library preparation and whole genome sequencing

The samples were sequenced using Illumina and Oxford Nanopore Technologies (ONT). The Illumina library was prepared with 2 × 150 bp paired-end fragments on an Illumina NovaSeq platform. Quality control of the reads was assessed using FastQC v0.11.9 (Andrews 2010). Subsequently, adapters were cut from the reads and quality filtered using Fastp v0.20.0 tool (Chen et al. 2018) with the following parameters: --detect_adapter_for_pe -f 12 -F 12. For genome sequencing using long reads, the Rapid Sequencing Kit SQK-RBK004 (ONT) was used for library preparation, and sequencing was performed using an R9.4 flow cell (FLO-MIN106D) on a MinION Mk1C machine (ONT) using MinKNOW v4.3.7 software. Basecalling was performed using Guppy v5.0.12, in the fast mode. Quality control of the reads was assessed using the Nanoplot v1.40.0 tool (De Coster et al. 2018). Subsequently, the adapters were cut with Porechop v0.2.4, and sequences with a quality equal to or greater than 10 were filtered out using Nanofilt v2.8.0 (De Coster et al. 2018).

### Hybrid genome assembly

To achieve *de novo* hybrid assembly, short and long reads that passed quality control and filtering were assembled using Unycicler v.0.4.8 tool (Wick et al. 2017). Subsequently, the hybrid genomes were polished with Medaka v1.2.3 (https://github.com/nanoporetech/medaka) using ONT reads. Then, the genomes were again polished with Polypolish v0.5.0 (Wick and Holt 2022) and poLCA v4.0.5 (Zimin and Salzberg 2020) using short reads. Finally, quality and contamination determination of the polished hybrid genomes were performed using Quast v5.0.2 and CheckM v1.1.3 (Gurevich et al. 2013; Parks et al. 2015). 

### Genomic and taxonomic annotation

Annotation of genomic sequences was achieved using Prokka v1.14 (Seemann 2014). The taxonomic annotation was performed by uploading the hybridized assembled genomes to the Type Strain Genome Server (TYGS) available under https://tygs.dsmz.de (Meier-Kolthoff and Göker 2019). Subsequently, the average nucleotide identity (ANI) was calculated using the FastANI v1.32 tool (Jain et al. 2018), and digital DNA-DNA hybridization (dDDH) was performed using the web tool Genome-to-Genome Distance Calculator (GGDC) (Meier-Kolthoff et al. 2022). In addition, a genome-based phylogenetic tree was inferred using the autoMLST: Automated Multi-Locus Species Tree pipeline (https://automlst.ziemertlab.com/), where the “denovo mode” was used and the IQ-TREE Ultrafast Bootstrap analysis (1000 replicates) option was selected (Alanjary et al. 2019). The constructed tree was visualized and annotated using the Interactive Tree Of Life (iTOL) available at https://itol.embl.de/ (Letunic and Bork 2021).

### Identification and analysis of biosynthetic gene clusters (BGCs)

The biosynthetic gene clusters in the nine strains were identified using antiSMASH v6.1.1 (Blin et al., 2021) with the following parameters: --taxon bacteria --fullhmmer --cc-mibig --cb-knownclusters --rre --hmmdetection-strictness strict and --genefinding-tool prodigal. Finally, to summarize the number of BGCs identified in the samples, a matrix plot was created using RAWGraphs (Mauri et al. 2017).

### Biosynthetic gene clusters network of the family *Micrococcaceae*

Genome assemblies belonging to the family *Micrococcaceae* were recovered from the Biosample database of the National Center for Biotechnology Information (NCBI), which includes the environmental packages of soil (50), plant-associated (43), water (11), sediments (7), and wastewater/sludge (4). Subsequently, the BGCs were identified using the same methodology described in this manuscript. To determine biosynthetic biodiversity, sequence similarity networks were generated using the “biosynthetic gene similarity clustering and prospecting engine” BiG-SCAPE v1.1.2 (Navarro-Muñoz et al. 2020) with the PFAM database (v35.0) (Mistry et al. 2021). A distance matrix was created by calculating the distance between every pair of BGCs within the dataset. This distance matrix incorporates three metrics: (i) Jaccard index, representing the percentage of shared domain types; ii) domain sequence similarity, reflecting the similarity between aligned domain sequences; and (iii) adjacency index, which measures the similarity of domain pair types. In addition, the MiBIG database v3.1 (Minimum Information about a Biosynthetic Gene cluster) (Terlouw et al. 2023) was used. The .gbk files of the BGCs obtained from antiSMASH were used as the inputs. The following settings were used to run the analysis: --mode auto --mix --no_classify --mibig --cutoffs 0.3 0.4. Finally, the networks obtained were annotated and visualized using Cytoscape v3.6.0 (Shannon et al. 2003).

## Results

### Antarctic isolates genome sequencing and taxonomic affiliation

We successfully isolated nine *Actinomycetota* strains from different soil and sediment samples from the Antarctic, following different sample treatments (Table 1). The hybrid assembly of those nine genomes, isolated from samples collected in the Antarctic territory, exhibited completeness exceeding 98% and contamination of less than 1%. The number of contigs varied between 1 and 5 for all assemblies, demonstrating a high quality of contiguity. Specifically, all assemblies had an L50 value of 1 and a notably high N50 value. The strain Sec 6.3 exhibited the lower contiguity. Furthermore, a large number of reads were obtained for each assembly, contributing to the genomic coverage and continuity (Supplementary Table S1). Additionally, all assemblies obtained had a guanine-cytosine content (GC) ranging from 61.5 to 72.9%, with predicted coding sequences numbering between 2348 and 5007, as expected for *Actinomycetota* phylum (Table 2).

**Table 1:** Origin and isolation description of nine bacterial strains obtained from soil and sediment samples from the Antarctic

Isolate strain	Geographical location	Sample source	Sample type	Pretreatment	Isolation medium
Soc 4.6	Antarctica: Deception Island	Deception soil	Soil	No	Oatmeal agar
Sec 5.1	Antarctica: Deception Island	Deception lagoon	Sediment	Phenol	Oatmeal agar
Sec 5.7	Antarctica: Deception Island	Deception lagoon	Sediment	No	Soil agar
Sec 5.8	Antarctica: Deception Island	Deception lagoon	Sediment	No	Soil agar
Sec 5.9	Antarctica: Deception Island	Deception lagoon	Sediment	SDS	Oatmeal agar
Sec 6.3	Antarctica: Doumer Island	Yelcho soil	Soil	Phenol	Soil agar
Sec 6.4	Antarctica: Doumer Island	Yelcho soil	Soil	Chloramine-T	Oatmeal agar
Sec 7.4	Antarctica: Doumer Island	Yelcho channel sediment	Sediment	No	Oatmeal agar
Se 16.17	Antarctica: Rey Jorge (King George) Island, Fildes Peninsula	Lake sediment	Sediment	Chloramine-T	Oatmeal agar

**Table 2:** Quality assessments and annotation of hybrid genome assemblies from nine Antarctic isolates

Strain	Genome length	Contigs	N50	L50	GC (%)	Completeness (%)	Contamination (%)	CDS	rRNA	tRNA	tmRNA
Soc 4.6	4,806,652	3	4,746,819	1	71.57	99.46	0	4407	6	52	1
Sec 5.1	5,426,886	1	5,426,886	1	61.49	99.12	0.39	5007	18	56	1
Sec 5.7	4,402,594	2	4,307,194	1	63.92	99.71	0	4215	12	55	1
Sec 5.8	3,870,955	1	3,870,955	1	66.14	99.71	0	3534	15	53	2
Sec 5.9	3,530,731	2	3,492,674	1	71.81	99.82	0.14	3493	6	50	1
Sec 6.3	2,568,479	4	1,294,273	1	72.88	98.21	0.23	2348	6	50	1
Sec 6.4	3,792,347	3	3,718,359	1	65.54	100	0.36	3579	6	49	1
Sec 7.4	4,384,395	5	3,981,032	1	66.81	99.71	0.49	3973	12	60	1
Se 16.17	4,883,589	4	4,495,321	1	62.67	99.71	0.73	4440	18	56	1

**Fig. 1:**
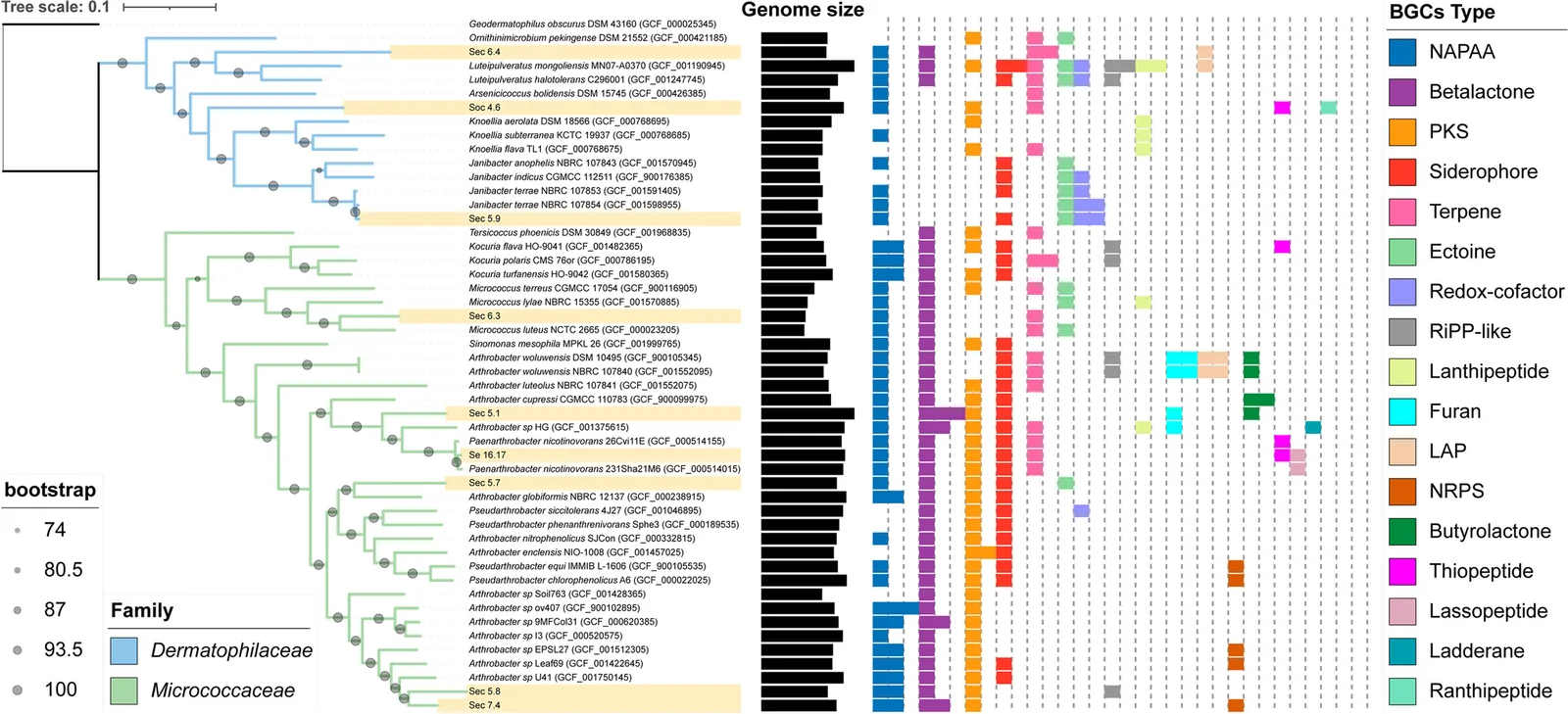
Multi-locus phylogenetic species tree indicating the distances between isolated strains (highlighted in yellow) and closely related species. The tree was constructed using the web tool autoMLST with the denovo mode and 1000 bootstrap replicates. Circles on branches indicate bootstrap values of > 74%. The Genome Taxonomy Database was used to obtain taxonomic information. *Geodermatophilus obscurus* DSM 43160 (GCF_000025345) was used as an outgroup. Bar charts in the middle represent the genome size of the strains in the tree. The barplot on the right side shows the number of BGCs of the species in the tree, where each color belongs to a type of BGCs according to the legend

The genomics-based tree revealed that the strains belonged to the families *Micrococcaceae* and *Dermatophilaceae* within the phylum *Actinomycetota*, as per the information sourced from the Genome Taxonomy Database (available at https://gtdb.ecogenomic.org/) (Figure 1). Specifically, within the family *Micrococcaceae*, strains Sec 5.8 and Sec 7.4 were closely grouped, and Sec 5.7 formed a distinct phylogenetic lineage within the genus *Arthrobacter*. On the other hand, strain Se 16.17 was grouped with *Paenarthrobacter nicotinovorans* 231Sha2.1M6, while Sec 5.1 formed a distinct phylogenetic lineage within the genus *Paenarthrobacter.* Strain Sec 6.3 was placed in a separate phylogenetic lineage within the genus *Micrococcus*. Additionally, strain Sec 5.9 was clustered with the species *Janibacter terrae*, and strains Sec 6.4 and Soc 4.6 formed a distinct phylogenetic lineage within the *Dermatophilaceae* family.

Comparing the whole genomic sequences using ANI and dDDH values, we found that almost all the strains exhibited the closest ANI values to species obtained through the NCBI database via autoMLST. However, these values were relatively low, except for strain Se 16.17, which shared a high similarity with *P. nicotinovorans* 231Sha2.1M6, showcasing ANI and dDDH values of 98.8% and 86.6%, respectively, and allowing its affiliation as a *P. nicotinovorans* strain. Another exception was strain Sec 5.9, showing 98.8% ANI and 90.1% dDDH, classifying it as the species *Janibacter terrae* (Table 3). Based on our results, seven out of nine strains might be representative of novel species of the genera *Lapillicoccus* sp. (Soc 4.6), *Pseudarthrobacter* sp. (Sec 5.7, Sec 5.8 and Sec 7.4), *Micrococcus* sp. (Sec 6.3), and *Allobranchiibius* sp. (Sec 6.4).

**Table 3:** Average nucleotide identity and digital DNA-DNA hybridization values between the isolated strains and their closely related species obtained by the Type Strain Genome Server (TYGS) and Automated Multi-Locus Species Tree (autoMLST)

Strain	Closest genome (TYGS)	ANI (%)	dDDH (%)	Closest genome (autoMLST)	ANI (%)	dDDH (%)
Soc 4.6	*Lapillicoccus jejuensis* DSM 18607	81.7	21.4	*Phycicoccus* sp. Root563	79.5	20.4
Sec 5.1	*Paenarthrobacter nicotinovorans* DSM 420	83.3	23.7	*Paenarthrobacter nitroguajacolicus* HG	85.3	24.3
Sec 5.7	*Pseudarthrobacter psychrotolerans* YJ56	81.8	23.6	*Arthrobacter* sp. KBS0703	82.9	23.2
Sec 5.8	*Pseudarthrobacter albicanus* NJ-Z5	83.2	25.4	*Arthrobacter* sp. U41	86.7	29.5
Sec 5.9	*Janibacter terrae* NBRC 107854	98.8	90.1	*Janibacter terrae* NBRC 107854	98.8	90.1
Sec 6.3	*Micrococcus endophyticus* BCRC 16908	88.7	33.6	*Micrococcus luteus* RIT305	88.9	25.5
Sec 6.4	*Allobranchiibius huperziae* DSM 29531	80.0	20.7	*Dermacoccus* sp. PE3	74.9	19.6
Sec 7.4	*Pseudarthrobacter albicanus* NJ-Z5	84.8	27.1	*Arthrobacter* sp. U41	89.5	35.5
Se 16.17	*Paenarthrobacter nitroguajacolicus* HG	85.2	27.2	*Paenarthrobacter nicotinovorans* 231Sha2.1M6	98.8	86.6

### Analysis and comparison of biosynthetic gene clusters

The nine genomes isolated from the *Micrococcaceae* and *Dermatophilaceae* families collectively possess 49 BGCs. Remarkably, about 84% of these BGCs exhibit a similarity of less than 50% with the closest clusters in the MiBIG database. The most prevalent BGC types within the strains were beta-lactones, type III polyketide synthases (T3PKS), and non-alpha-poly amino acids (NAPAA), with all of them containing NAPAA. Furthermore, BGCs corresponding to siderophores, terpenes, thiopeptide, and non-ribosomal peptide synthetase (NRPS), among others, were identified in smaller quantities (Supplementary Figure S1).

Among the BGCs exhibiting a similarity identity greater than 50% with known clusters in the database, we found siderophores with 75% and 100% similarity to the desferrioxamine E cluster; a type III polyketide synthase with 100% similarity to the alkylresorcinol; a cluster with 75% similarity to the ectoine; and a terpene from strain Sec 6.3, showing 66% similarity to the carotenoid cluster (Supplementary Figure S2). The strains belonging to the *Micrococcaceae* family shared NAPAA, beta-lactone, and T3PKS clusters. Then, we wanted to further investigate whether this feature could be associated with the taxonomic family or could be part of genetic material related to strains inhabiting the extreme environment.

### Biosynthetic gene cluster network analysis in the *Micrococcaceae* family

**Fig. 2:**
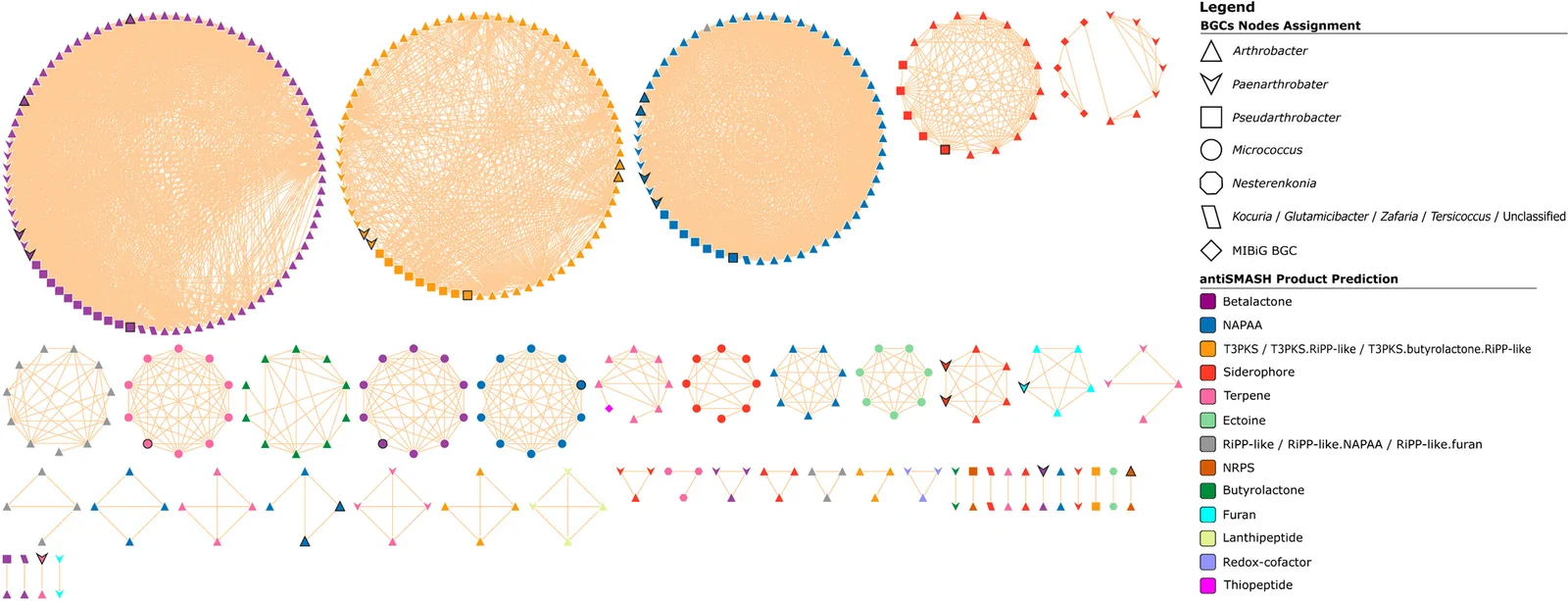
Sequence similarity network of 597 BGCs with a cutoff of 0.3 belonging to the *Micrococcaceae* family generated by BiG-SCAPE and visualized with Cytoscape v3.9.1. Each node represents an individual BGC, colored according to the antiSMASH product prediction. The nodes with black borders represent the BGCs identified in our Antarctic strains. Singletons, which are unique BGCs with no connections, are not displayed. NAPAA, non-alpha-polyamino acids; T3PKS, type iii polyketide synthases; RiPP, ribosomally synthesized and post-translationally modified peptides; NRPS, non-ribosomal peptide synthetase

To assess the biosynthetic biodiversity of the *Micrococcaceae* family, we employed the biosynthetic gene similarity clustering and prospecting engine (BiG-SCAPE) to generate sequence similarity networks. The analysis involved 597 BGCs identified from 121 genomic assemblies within the *Micrococcaceae* family (Fig. 2 and Supplementary Table S2). Among these, six assemblies were from strains isolated in Antarctica, while the rest were retrieved from the Biosample database of the NCBI. These genomes were classified under different environmental categories, including soil (50), plant-associated (43), water (11), sediments (7), and wastewater/sludge (4). The similarity cutoff used for the clustering was set at 0.3.

The sequence similarity network of the BGCs unveiled six predominant types: beta-lactone, NAPAA, T3PKS, siderophores, terpenes, and ectoine. Moreover, the networks containing BGCs from the genera *Micrococcus* and *Nesterenkonia* were distinctly grouped, unlike other genera that shared their BGCs within the same network. Within the identified clusters, two were noteworthy for their annotations from six MiBIG reference BGCs with known functions. Five of these were associated with siderophores, encompassing natural products such as desferrioxamine B (MIBiG BGC0000940 and BGC0000941), desferrioxamine E (MIBiG BGC0001478), and legonoxamine A (MIBiG BGC0002305). Conversely, one was linked to the thiopeptide category but grouped in the same cluster as terpenes, representing a compound called “TP-1161” (MIBiG BGC0000615). This limited representation of networks containing previously characterized BGCs underscores the extensive BGC diversity within the *Micrococcaceae* family. We attempted to generate networks based on the source of isolation and continent, but no discernible relationships were identified related to the origin of the isolated (Supplementary Figure S3 and S4).

## Discussion

The Antarctic stands as the coldest, driest, windiest, and most challenging-to-access continent on Earth. Due to its extreme conditions, it has been identified as a potential source for the discovery of novel natural products from bacteria (Núñez-Montero and Barrientos 2018). We isolated and analyzed nine strains from soil and sediment samples collected across diverse locations within the Antarctic territory. These strains were obtained through selective isolation methods to increase the probability of obtaining rare strains and to facilitate the discovery of natural products. Heating in the pretreatment of the samples, the use of chemicals such as phenol and sodium dodecylsulfate and antimicrobial agents, such as nalidixic acid, was used to reduce the growth of undesirable bacteria and increase the selectivity of rare *Actinomycetota* strains. This is possible because there are several strains of *Actinomycetota* resistant to a broad spectrum of antibiotics, together with the addition of chemical compounds toxic to bacteria, which allows for a decrease in the number of bacteria sensitive to these agents, thus enabling the isolation of new strains for the search of new natural products (Subramani and Aalbersberg 2013).

Genomic assembly of the isolated strains was facilitated using a hybrid approach, integrating short reads through Illumina sequencing and long reads using Oxford Nanopore Technology (ONT). Combining these sequencing technologies enhances genome assembly, addressing the inherent limitations of Illumina, which struggles with errors in sequencing repetitive structures and relies on short read sizes, typically less than 500 base pairs. These challenges are mitigated with ONT, generating large reads, often exceeding 10,000 bp. This allows for the assembly of complex genomes, resulting in fewer contigs and enhanced precision and contiguity (De Maio et al. 2019). This is evidenced by genomic annotation, wherein all assemblies display a high N50 value and a low L50 value, except for strain Sec 6.3, which demonstrates a slightly lower N50 value. The L50 and N50 values represent the number of contigs and contig lengths, respectively, crossing the 50% mark of the assembly. Hence, higher N50 values and lower L50 values signify a more contiguous assembly (Jayakumar and Sakakibara 2019). Additionally, we achieved a substantial depth of coverage in the genomic assemblies utilizing both technologies, as outlined in the supplementary tables. Consequently, this precise analysis allows for a thorough examination of the biosynthetic gene clusters and various genomic characteristics of the obtained strains.

Taxonomic annotation revealed that strains Sec 5.9 and Se 16.17 exhibited high dDDH and ANI values, with their closest genomes being *Janibacter terrae* NBRC 107854 and *Paenarthrobacter nicotinovorans* 231Sha2.1M6, respectively. The generally accepted cutoff values for identifying new species within a genus are 95–96% for ANI and AAI and 70% for dDDH (Richter and Rosselló-Móra 2009; Chun et al. 2018). Consequently, these strains are identified as belonging to the mentioned species. Strains affiliated with the *J. terrae* species were previously isolated from environmental water and soil samples, showcasing the capability to degrade environmental pollutants (Lang et al. 2003). On the other hand, *P. nicotinovorans 231Sha2.1M6* was isolated from *Arabidopsis thaliana* soil samples. As for the remaining seven strains, both their dDDH and ANI values fell below the defined cutoff values, designating them as new species within the *Micrococcaceae* and *Dermatophilaceae* families.

In the analysis of the identified BGCs within the isolated strains, a variable number, ranging from three to eight BGCs, was observed. Among them, three main types stood out: NAPAA, beta-lactone, and T3PKS. Beta-lactone-associated BGCs have demonstrated applications as anticancer agents, such as Marizomib (salinosporamide A) and antimicrobial and antiobesity agents, like Xenical (Robinson et al. 2019; Wang et al. 2021a, b). T3PKS, responsible for biosynthesizing a plethora of natural products, encompass antibiotics, immunosuppressants, and cancer chemotherapy (Nivina et al. 2019). NAPAA, such as ε-poly-L-lysine, exhibit bacteriostatic and biodegradable properties, leading to their use as food preservatives and in the pharmaceutical industry (Wang et al. 2021a, b). Another noteworthy category of BGCs with potential public health applications includes RiPPs, which feature a domain known as the RiPP recognition element (RRE-containing). This domain is prevalent in various RiPP clusters (Kloosterman et al. 2020) and has been instrumental in addressing therapeutic challenges, such as combating cystic fibrosis and acting as antimicrobials (Hetrick and Donk 2017).

Furthermore, concerning BGCs with a similarity greater than 50% to known BGCs in the MiBIG database, we identified alkylresorcinol in the Soc 4.6 strain. Alkylresorcinol is a lipophilic molecule with a polyphenol structure known for its antimicrobial, anticancer, antilipidemic, antioxidant, and other properties (Zabolotneva et al. 2022). Additionally, we found BGCs linked to siderophores, particularly desferrioxamine E in the Sec 5.1, 5.7, 5.9, and Se 16.17 strains, which play a crucial role in microbial growth (Yamanaka et al. 2005). On a different note, we also identified BGCs associated with more physiological functions, such as terpenes and ectoine. Terpenes are linked to electron transport, light uptake, photoprotection, and signaling (Caulier et al. 2019), while ectoine is known for its protective properties on enzymes, DNA, cell membranes, and cells against various types of stress, including osmotic, cold, and heat stress (Zhang et al. 2009). Hence, our results showed that the possibly known molecules are related to primary microbial metabolism and or common functions across. On the other hand, our strains showed a large number of BGCs with low or non-similarity to other clusters, and belonging to types of specialized metabolites previously related to biological activities. Hence, our data highlights the metabolic potential for the production of novel natural products from novel strains isolated from the Antarctic continent.

The network analysis of BGCs allows us to visualize a phylogenetic correlation between certain genera of the family *Micrococcaceae*, especially observed in genera such as *Micrococcus* and *Nesterenkonia*. This observation has been observed in studies of other species belonging to the phylum *Actinomycetota*, such as the genera *Amycolatopsis*, *Salinispora*, and *Streptomyces*, among others, where these correlations were detected using different bioinformatic approaches, such as the identification of gene cluster families, operational biosynthetic units, and BGCs (Doroghazi and Metcalf 2013; Ziemert et al. 2014; Adamek et al. 2018). In addition, it has been detected in the phylum Firmicutes, with the genus *Bacillus*, where clade- and species-specific BGCs have been found (Steinke et al. 2021). These BGCs-related families might be associated with core metabolism of the genus. Furthermore, among the three most abundant groups of BGCs within this family, T3PKS have been previously reported for the genus *Arthrobacter* (Doroghazi and Metcalf 2013), but not the beta-lactone BGCs, which are grouped in one network and currently do not have a close reference and could be part of the core genome of these strains because of their high level of conservation.

In addition, no relationship was found between the source of isolation and the BGCs. This might be because there are many types of environments and different conditions of temperature, humidity, and pressure, among others, which generates a very diverse grouping in the network. On the other hand, there are many BGCs to be studied within this family, since only two groups are similar to any known natural product, being siderophores and thiopeptides. The latter obtained from an *Actinomycetota*, *Nocardiopsis* sp., has potent antibiotic properties (Engelhardt et al. 2010). Therefore, it is important to further investigate BGCs belonging to this family as several beneficial properties have been demonstrated, such as the ability to act as plant growth promoters in various plant species, as seen in members belonging to the genera *Arthrobacter* and *Pseudarthrobacter* (Chhetri et al. 2022; Jiang et al. 2022; Ham et al. 2022; Platamone et al. 2023). In addition, species of the genera *Arthrobacter* and *Micrococcus* have been used for the bioremediation of organic compounds and heavy metals (Behera and Das 2023). For the species belonging to the genus *Micrococcus*, extracts with pharmaceutical properties, such as antimicrobial, antifungal, and antioxidant properties, have been obtained, which are described in more detail in the review by Tizabi and Hill (2023).

In summary, the species isolated and sequenced in this study exhibit BGCs with significant potential across various domains including clinical applications, pharmaceuticals, the food industry, and agriculture. Moreover, we observed a substantial gap in our understanding of the functionality of different BGC groups within the *Micrococcaceae* family, which holds significant implications for the future. Our research also provides valuable genomics data from untapped microorganisms from rare *Actinomycetota* strains from extreme environments.

Given that this analysis was conducted *in silico*, we were unable to confirm whether these BGCs are actively expressed or silenced. Future investigations integrating transcriptomics and metabolomics are essential to determine their expression and functionality. This comprehensive approach will pave the way for these BGCs to emerge as promising candidates for future applications and biotechnological advancements.

## Supplementary Information

Below is the link to the electronic supplementary material.

Supplementary file1 (TIF 28338 KB)

Supplementary file2 (PDF 615 KB)

Supplementary file3 (XLSX 36 KB)

## Data Availability

The microbial reads and genome assemblies generated in this study are available in the National Center for Biotechnology Information (NCBI) under the BioProject ID: PRJNA1063326.
