# Supplementary material for: Biosynthetic gene clusters with biotechnological applications in novel Antarctic isolates from Actinomycetota
Source: Appl Microbiol Biotechnol. 2024 May 8;108:325. doi: 10.1007/s00253-024-13154-x (PMC11078813; doi:10.1007/s00253-024-13154-x)
Supplement: Supplementary file 1 [file 253_2024_13154_MOESM2_ESM.pdf]

Supplementary Information

Biosynthetic Genes Clusters with Biotechnological Applications in Novel Antarctic isolates from *Actinomycetota*

Pablo Bruna[1, 2], Kattia Núñez-Montero[3,4], María José Contreras[5], Karla Leal[5], Matías García[1,2,6], Michel Abanto[2]*, Leticia Barrientos[3]*

1 Programa de Doctorado en Ciencias mención Biología Celular y Molecular, Universidad de La Frontera.

2 Núcleo Científico y Tecnológico en Biorecursos (BIOREN), Universidad de La Frontera, Avenida Francisco Salazar, 01145, Temuco, Chile.

3 Facultad de Ciencias de la Salud, Instituto de Ciencias Aplicadas, Universidad Autónoma de Chile, Avenida Alemania 1090. Temuco, Chile.

4 Centro de Investigación en Biotecnología, Departamento de Biología, Instituto Tecnológico de Costa Rica, Cartago, Costa Rica

5 Facultad de Ingeniería, Instituto de Ciencias Aplicadas, Universidad Autónoma de Chile, Avenida Alemania 1090. Temuco, Chile.

6 Laboratorio de Biocontrol, Facultad de Ciencias Agropecuarias y Medioambiente, Universidad de La Frontera, Temuco Chile.

*Corresponding author: mfabanto@gmail.com, leticia.barrientos@uautonoma.cl

Supplementary Table S1. Data obtained from reads sequenced by Illumina and Oxford Nanopore Technologies.

	**MinION long reads**				**Illumina NovaSeq short reads**	
**Strain**	**Read length N50 (bp)**	**Mean read quality (Q)**	**Number of reads**	**Total bases pairs (bp)**	**Total reads (M)**	**Total bases (G)**
Soc 4.6	8,040	12.9	58,966	293,288,025	12.198648	1.683066
Sec 5.1	6,077	11.4	220,073	838,364,944	12.825206	1.769476
Sec 5.7	7,516	12.1	329,207	1,595,242,570	15.512778	2.140573
Sec 5.8	17,881	11.7	87,149	929,874,256	15.901406	2.193913
Sec 5.9	6,957	11.5	236,238	867,710,676	9.021834	1.244746
Sec 6.3	5,498	12.0	249,604	928,547,984	14.284136	1.970608
Sec 6.4	5,865	13.0	276,355	1,056,143,791	13.034698	1.798543
Sec 7.4	10,539	12.2	31,421	214,346,230	8.872462	1.224215
Se 16.17	6,301	12.0	312,723	1,306,975,517	10.283564	1.418739

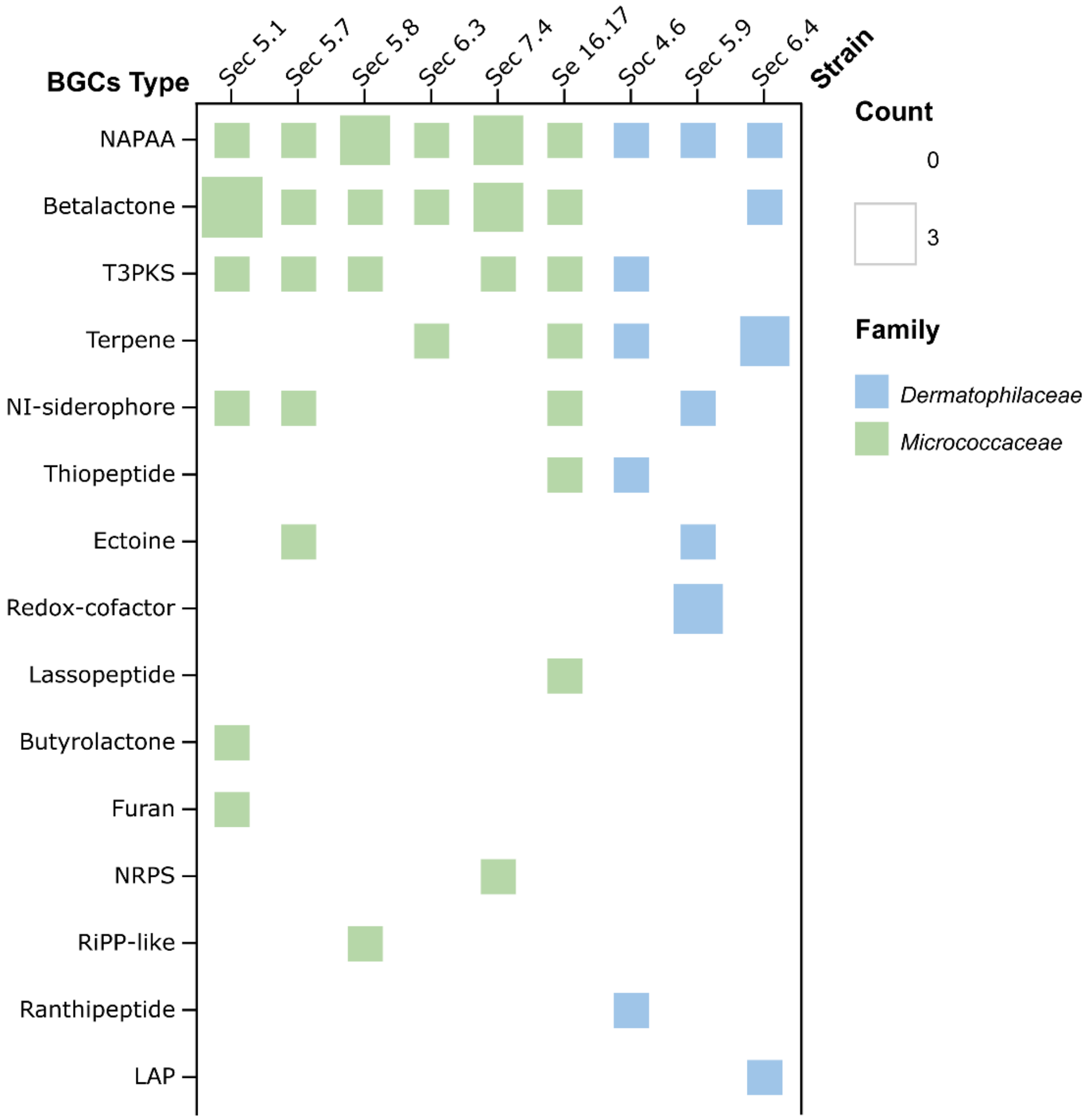

Supplementary Figure S1. Number of biosynthetic gene clusters detected by antiSMASH in the isolated strains. Green and blue light colors refer to taxonomic annotations based on the family using the Genome Taxonomy Database. The size of the squares indicates the number of BGCs.

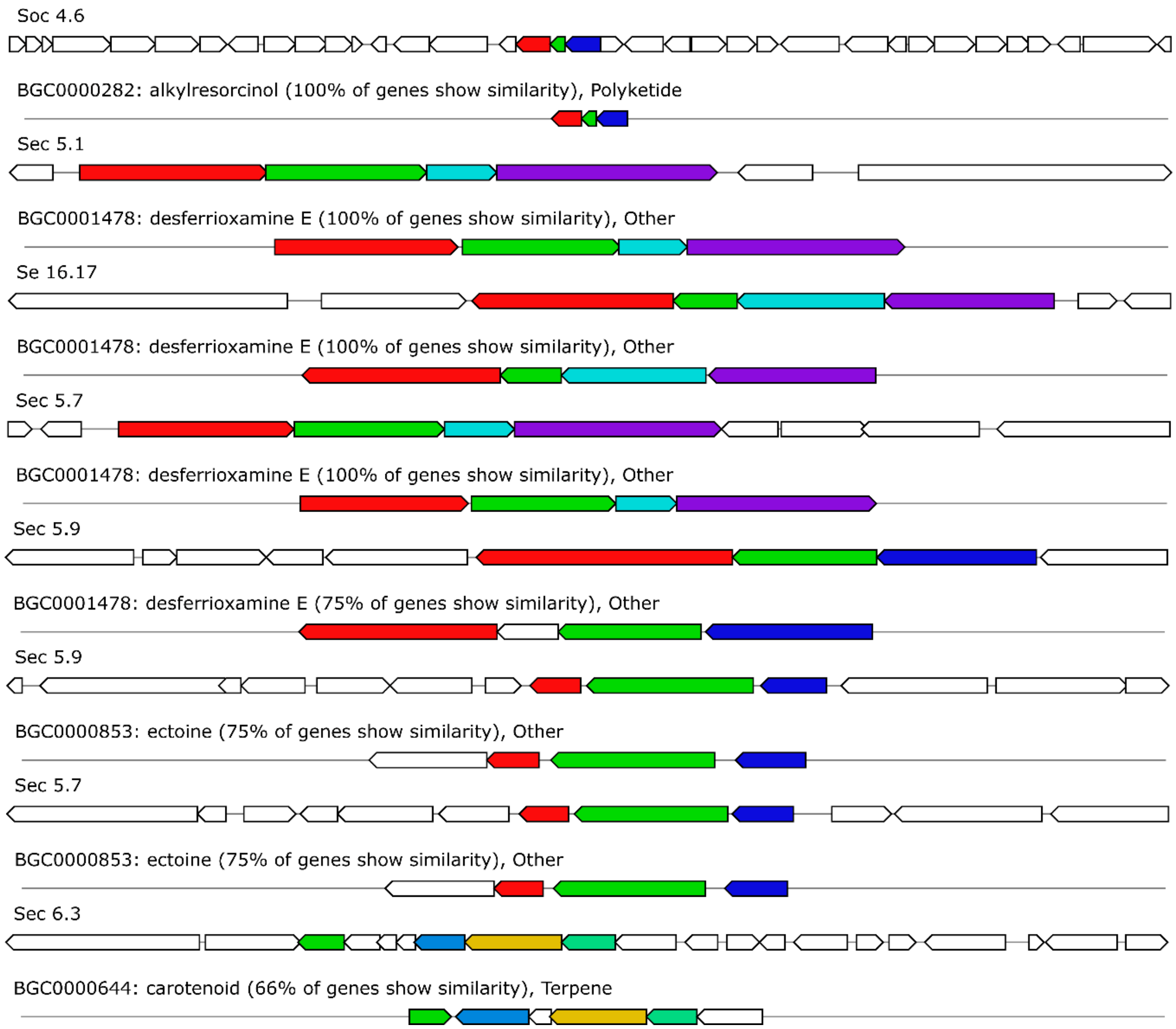

Supplementary Figure S2. Analysis of the identified biosynthetic gene clusters (BGCs) and their similarities to known clusters. BGCs that exhibited similarity identities greater than 50% with known clusters in the database were visualized. Red, green, cyan, blue, purple, and yellow highlight the specific gene correspondences involved in the biosynthesis of recognized metabolites.

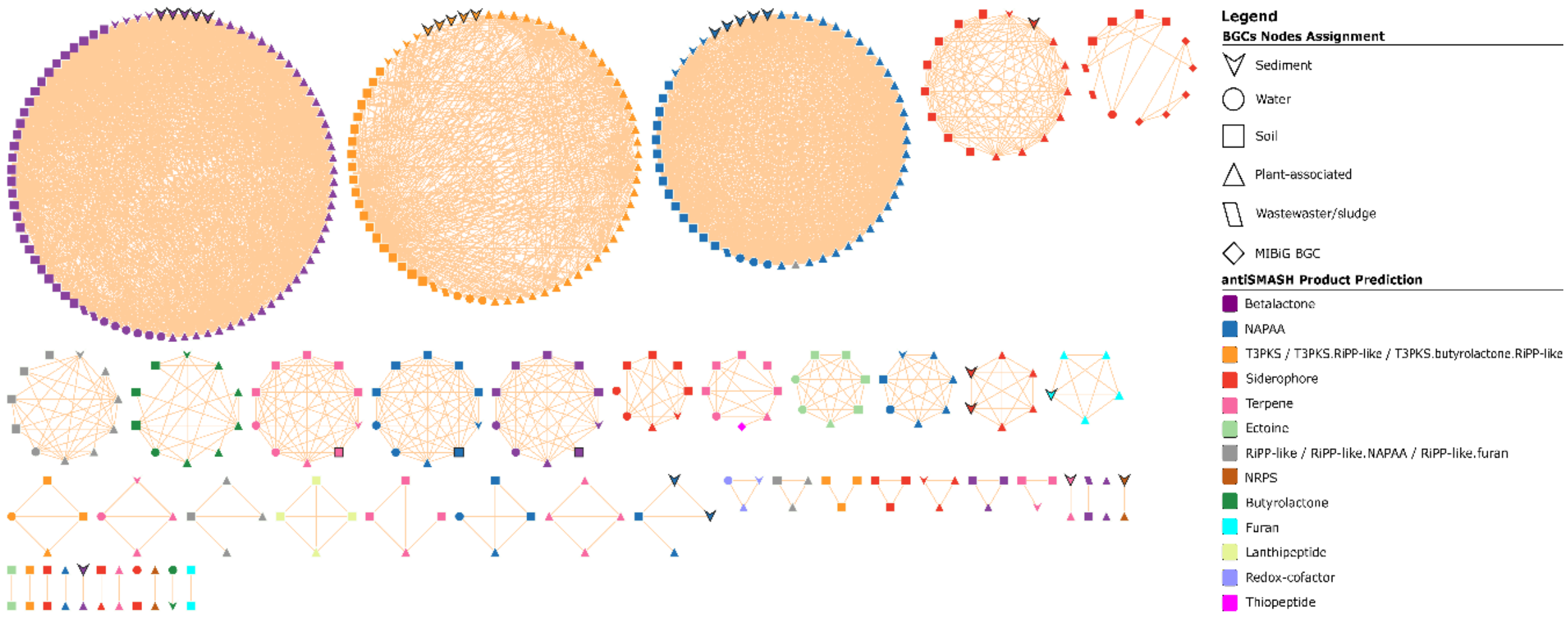

Supplementary Figure S3. Sequence similarity network of 597 BGCs with a cutoff of 0.3 belonging to the *Micrococcaceae* family generated by BiG-SCAPE and visualized with Cytoscape v3.9.1. Each node represents an individual BGC, colored according to the antiSMASH product prediction. The nodes with black borders represent the BGCs identified in our Antarctic strains. Singletons, which are unique BGCs with no connections, are not displayed. NAPAA, non-alpha-polyamino acids; T3PKS, type iii polyketide synthases; RiPP, ribosomally synthesized and post-translationally modified peptides; NRPS, non-ribosomal peptide synthetase. The assignment of nodes is related to the source of isolation metadata obtained from the Biosample database of the National Center for Biotechnology Information.

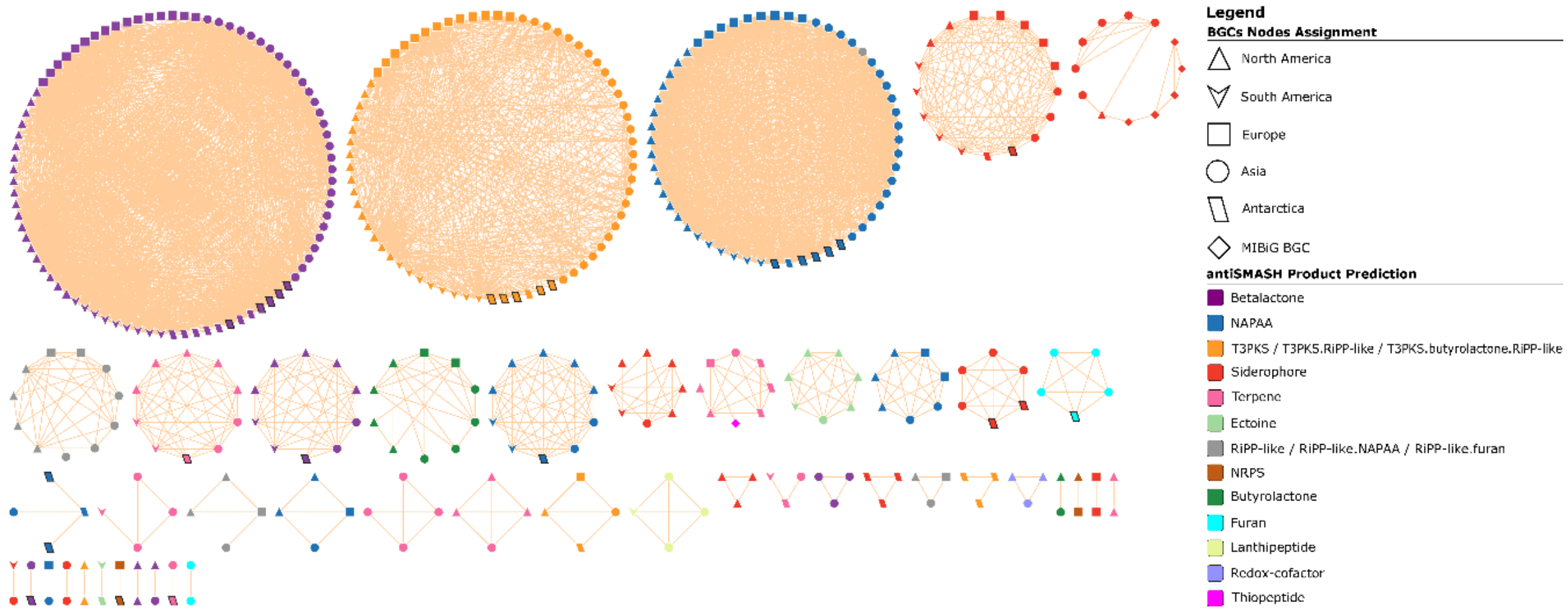

Supplementary Figure S4. Sequence similarity network of 597 BGCs with a cutoff of 0.3 belonging to the Micrococcaceae family generated by BiG-SCAPE and visualized with Cytoscape v3.9.1. Each node represents an individual BGC, colored according to the antiSMASH product prediction. The nodes with black borders represent the BGCs identified in our Antarctic strains. Singletons, which are unique BGCs with no connections, are not displayed. NAPAA, non-alpha-polyamino acids; T3PKS, type iii polyketide synthases; RiPP, ribosomally synthesized and post-translationally modified peptides; NRPS, non-ribosomal peptide synthetase. The assignment of nodes is related to the geographic location metadata obtained from the Biosample database of the National Center for Biotechnology Information.
