# Supplementary figures and images for: Biosynthetic gene clusters with biotechnological applications in novel Antarctic isolates from Actinomycetota
Source: Appl Microbiol Biotechnol. 2024 May 8;108(1):325. doi: 10.1007/s00253-024-13154-x (PMC11078813; doi:10.1007/s00253-024-13154-x)

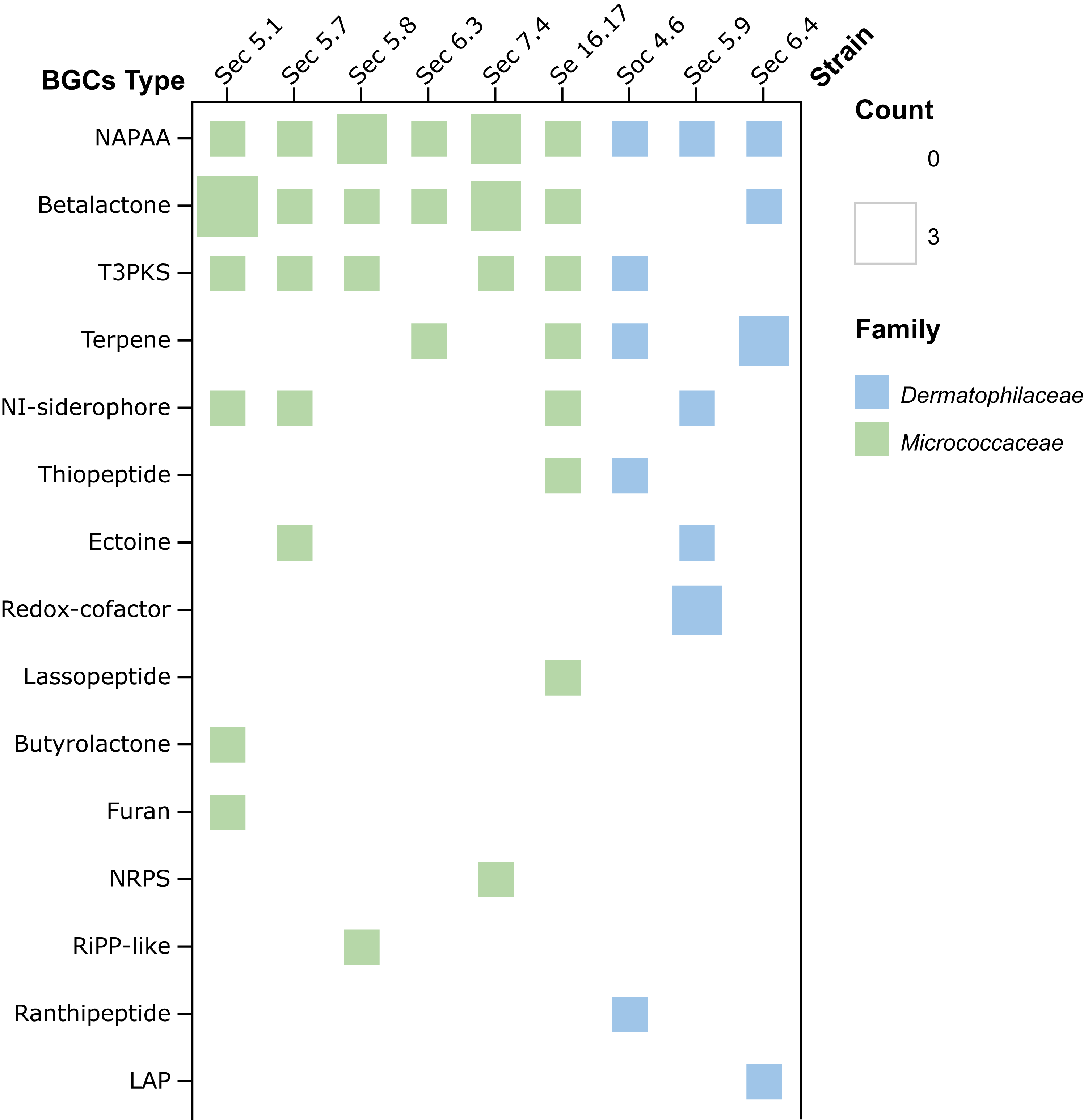

Supplement: Supplementary file 1 — Supplementary file1 (TIF 28338 KB) [file 253_2024_13154_MOESM1_ESM.tif]
